# Prevention and treatment of gerbil hepatitis E using the programmable CRISPR-Cas13d system

**DOI:** 10.1016/j.gendis.2023.06.020

**Published:** 2023-07-24

**Authors:** Chengcheng Zhao, Chong Li, Sa Li, Hanyu Wu, Pengyao Ren, Tianlong Liu, Xiaoxiang Hu, Ran Zhang

**Affiliations:** a State Key Laboratory of Animal Biotech Breeding, College of Biological Sciences, China Agricultural University, Beijing 100193, China; b Laboratory of Veterinary Pathology and Nanopathology, College of Veterinary Medicine, China Agricultural University, Beijing 100193, China

In recent years, the global incidence of hepatitis E virus (HEV) has been rising, leading to increased morbidity and mortality associated with hepatitis. Cas13, a CRISPR effector, shows promise as an antiviral agent against single-stranded RNA viruses. Cas13d, a type VI-D effector, exhibits higher efficiency in suppressing RNA viruses compared to other type VI variants. However, its *in vivo* activity against RNA viruses in mammals remains unknown. This study aimed to evaluate Cas13d′s effectiveness against HEV. By comparing HEV3 genotypes, we designed eight crRNAs targeting conserved regions, achieving a maximum interference efficiency of 94.82% using HEK293T cells. To enable *in vivo* targeting of HEV by Cas13d, we engineered synthetic AAV8 vectors for Cas13d delivery, along with efficient crRNAs. In gerbils, intraperitoneal injection of Cas13d and HEV3-targeting crRNAs packaged in AAV8 resulted in a 50% reduction in the detoxification rate and significantly suppressed incidence. Our study establishes an effective platform for preventing and treating HEV infection in gerbils, demonstrating the potential of CRISPR/Cas13d as an RNA-guided therapy against mammalian viruses beyond HEV.

Hepatitis E is a globally significant infectious disease caused by the HEV, primarily transmitted through the fecal-oral route. It leads to hepatitis symptoms, affecting millions and killing thousands of individuals.^1^ It can infect humans, pigs, and rabbits, which can then transmit it to humans. While antiviral therapeutics and vaccines exist, the need for more effective treatments against HEV remains. Cas13, an RNA-targeting CRISPR/Cas effector, has shown promising antiviral activity by specifically binding to and cleaving RNA. It has been observed to cleave RNA in bacterial systems and has the potential for multiplexed targeting. Cas13d, a specific type of Cas13 effector, is particularly suitable for delivery using AAV vectors.^2^ We aligned four HEV3 genomes to identify conserved regions for Cas13d targeting, resulting in eight target sites (HEV-crRNA1–8) ranging from 22 to 30 nt. Eight crRNAs were synthesized and constructed in the U6 promoter-driven expression vector, with one non-target crRNA selected as a control (Fig. S1A up). To evaluate viral mRNA knockdown, mCherry reporter systems were created using the mCherry fluorescence gene fusion GDC9, representing HEV3. Cas13d expression, driven by the CMV promoter, was transfected alongside reporter and crRNA expression vectors into HEK293FT cells on a 24-well plate (Fig. S1A down). After 48 h, the relative expression of crRNA1, crRNA2, crRNA3, and crRNA6 significantly decreased (*P* < 0.0001) compared with the control group. Fluorescence intensity indicated efficient suppression of the mCherry-fused ORF (Fig. 1A, B; Fig. S1B, C). Subsequently, crRNA1/2/3/6 were selected as primary targets for further experiments. We identified target sites in the HEV genome and demonstrated the ability of Cas13d to effectively interfere with HEV replication *in vitro*.

**Figure 1:**
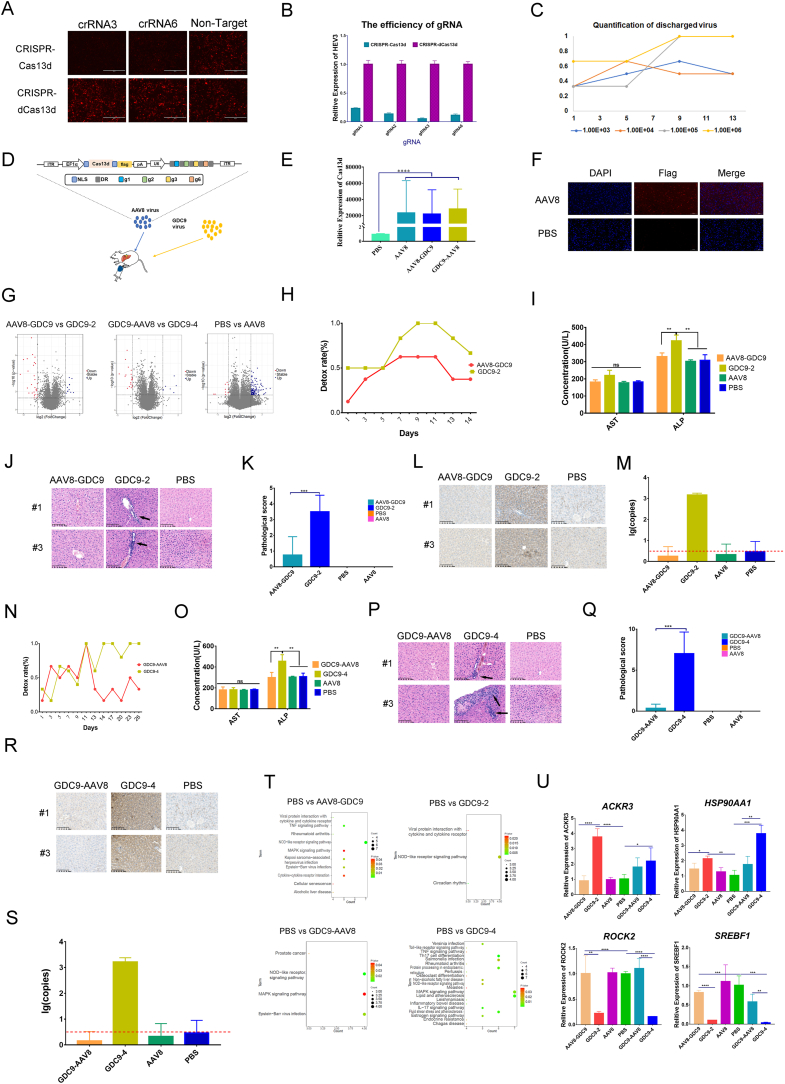
CRISPR-Cas13d system for the prevention and treatment of hepatitis E in gerbils. **(A)** mCherry expression in HEK293FT cells transfected with the mCherry-open reading frame (ORF) construct, Cas13d orthologs, and CRISPR RNAs (crRNAs). Non-Target, non-targeting crRNA. Scale bar: 400 μm. **(B)** mCherry-ORF mRNA relative expression. The data were shown as mean ± SD (*n* = 3; *P* < 0.0001). **(C)** Quantified detoxification after injecting gerbils with different viral doses. **(D)** Schematic illustration of intraperitoneal injection of AAV8 packing Cas13d-crRNA. **(E)** Cas13d mRNA expression in the liver of Mongolian gerbils after injection with AAV8. The data were shown as mean ± SEM (*n* = 4; *P* < 0.0001). **(F)** Representative Cas13d immunofluorescence staining of liver sections from Mongolian gerbils injected with AAV8. Scattered fluorescence was observed after injection with AAV8. Scale bar: 50 μm. **(G)** Volcano plot of differential transcript levels between groups (*n* = 3). AAV8 packing Cas13d-crRNA-GDC9: AAV8 packing Cas13d-crRNA injection first, followed by GDC9 injection; GDC9-2: GDC9 injection for only two weeks; GDC9-AAV8 packing Cas13d-crRNA: GDC9 injection first, followed by AAV8 packing Cas13d-crRNA injection; GDC9-4: HEV3 injection for only four weeks; PBS: PBS injection only. **(H)** Detection of detoxification after injection of gerbils in different groups. **(I)** Serum biochemical assays for detecting alkaline phosphatase (ALP) and aspartate aminotransferase (AST) reflecting hepatic damage and necrosis. The data were shown as mean ± SD (*n* = 4; *P* < 0.001). **(J)** Liver histology of gerbils injected with or without AAV8 packing Cas13d-crRNA and GDC9. The black arrowhead indicates vacuoles. Scale bar: 100 μm. **(K)** Liver pathological scoring of Mongolian gerbils injected with or without AAV8 packing Cas13d-crRNA and GDC9 (*n* = 4). Liver lesions were scored with 1 for mild inflammation, 2 for moderate inflammation, 3 for moderate inflammation with scattered necrosis, and 4 for moderate inflammation with aggregative and distinct necrosis. The grading was done by three pathologists who were blinded to the experiment. **(L)** GDC9 immunohistochemical staining of liver sections from gerbils injected with or without the AAV8 packing Cas13d-crRNA and GDC9 (*n* = 4). The darker the attachment color, the higher the virus content. Scale bar: 100 μm. **(M)** GDC9 copies significantly decreased after intraperitoneal injection of AAV8 packing Cas13d-crRNA, but injection of GDC9 alone did not result in such a decrease. AAV8 packing Cas13d-crRNA-GDC9: AAV8 packing Cas13d-crRNA injection first, followed by GDC9 injection. GDC9-2: GDC9 injection for only two weeks; AAV8: only AAV8 packing Cas13d-crRNA injection; PBS: only PBS injection. **(N)** Detection of detoxification after injection. **(O)** Serum biochemical assays of ALP and AST. The data were shown as mean ± SD (*n* = 4; *P* < 0.001). **(P)** Liver histology of gerbils injected with or without AAV8 packing Cas13d-crRNA and GDC9. The black arrowhead indicates vacuoles. Scale bar: 100 μm. **(Q)** Liver pathological scoring of Mongolian gerbils injected with or without AAV8 packing Cas13d-crRNA and GDC9 (*n* = 4). **(R)** Representative GDC9 immunohistochemical staining of liver sections from gerbils injected with or without the AAV8 packing Cas13d-crRNA and GDC9 (*n* = 4). The darker the attachment color, the higher the virus content. Scale bar: 100 μm. **(S)** GDC9 copies significantly decreased after intraperitoneal injection of AAV8 packing Cas13d-crRNA, but injection of GDC9 alone did result in such a decrease. GDC9-AAV8 packing Cas13d-crRNA: GDC9 injection first, followed by AAV8 packing Cas13d-crRNA injection; GDC9-4: GDC9 injection for only 4 weeks; AAV8: only AAV8 packing Cas13d-crRNA injection; PBS: only PBS injection. Note that the gerbils in PBS-1 to PBS-4 in J/P and L/R are the same, thus images of PBS-1 to PBS-4 are the same in both figures. **(T)** KEGG analysis of liver transcriptome data (*n* = 4). **(U)** Expression of DEGs via real-time fluorescence quantitative PCR. Gerbil liver samples (*n*) = 4. ^∗^*P* < 0.05, ^∗∗^*P* < 0.01, ^∗∗∗^*P* < 0.001, ^∗∗∗∗^*P* < 0.0001.

Antiviral properties of CRISPR-Cas13d were then evaluated in gerbils. Different doses of the HEV3-GDC9 virus were injected intraperitoneally (Fig. S2A; Fig. 1C), and the 5E+5 copies/mL treatment exhibited efficient detoxification. Liver lesions were observed, with the degree of inflammation and necrosis comparable to the 5E+6 copies/mL treatment (Fig. S2B). Cas13d delivery is crucial for its therapeutic application. Lentivirus-mediated delivery can prevent RNA virus spread, but the exogenous gene introduction raises safety concerns. AAV delivery is a viable option, and Cas13d′s small size allows it to be packaged with a CRISPR array encoding multiple guide RNAs. Subsequently, AAV8 and AAV rh10 were used as liver-targeted gene delivery systems. GFP mRNA expression increased with the injection dose and time (Fig. S2D–E), with AAV8 demonstrating higher delivery efficiency than AAV rh10. AAV8 with a dose of 3 E+12 GC/mL was selected for further experiments. AAV8 successfully delivered Cas13d to gerbil liver cells, as confirmed by Cas13d mRNA expression and immunofluorescence analysis (Fig. 1D–F). This study established an antivirus platform utilizing AAV8 to deliver Cas13d and a specific HEV crRNA sequence.

The specificity of Cas13d was examined through RNA-seq analysis, revealing no significant off-target effects (Fig. 1G). Cas13d-mediated transcriptional interference was shown to be safe and efficient in mammals. Compared with other RNA interference techniques,^3^ Cas13d-mediated knockdown exhibited similar or higher efficiency with significantly fewer off-target effects. This makes Cas13d a promising candidate for antiviral therapy without the risk of permanent DNA alterations associated with standard CRISPR-Cas9 editing. Our study showed no significant off-target effects on the host transcriptome when targeting the virus with Cas13d. To test the antiviral effect of Cas13d, gerbils were injected with AAV8 packing Cas13d: crRNA and subsequently challenged with the GDC9 virus (Fig. S3A). AAV8 packing Cas13d: crRNA significantly inhibited HEV infection, as indicated by the detoxification rate (Fig. 1H) and liver damage markers (Fig. 1I). Histological analysis demonstrated reduced hepatic inflammation and necrosis in gerbils injected with AAV8 packing Cas13d: crRNA compared with the GDC9-only group (Fig. 1J, K; Fig. S3B). Immunohistochemical analysis confirmed a lower viral load in the AAV8 packing Cas13d:crRNA group (Fig. 1L, M; Fig. S3C). Intraperitoneal injection of Cas13d in gerbils successfully suppressed viral RNA, indicating its potential for both prevention and treatment of HEV infection.

Intraperitoneal injection of AAV8 packing Cas13d-crRNA was then investigated as a potential treatment for hepatitis E infection (Fig. S4A). A high detoxification rate was observed in the GDC9-only group, which decreased sharply in the AAV8 packing Cas13d-crRNA group after 11 days (Fig. S1N). Liver function tests and histology revealed similar results between AAV8 packing Cas13d-crRNA-injected gerbils and control groups, indicating the safety and efficacy of the Cas13d system in treating hepatitis E (Fig. 1O–Q; Fig. S4B). Immunohistochemical analysis showed a significantly lower viral load in the AAV8 packing Cas13d-crRNA group (Fig. 1R, S; Fig. S4C). With no FDA-approved therapies for HEV and challenges in vaccine efficacy and drug resistance, novel antiviral strategies are needed. Programmable antiviral technologies such as the Cas13d system enable the rapid development of therapeutics targeting various pathogens. The efficacy of Cas13d has been demonstrated in targeting different viruses in plants and fish,^4^ making it a versatile and specific antiviral platform.

This study also explored the mechanism by which the Cas13d system alleviates liver injury. Analysis of liver transcriptome data highlighted pathways such as NOD-like receptor signaling, viral protein interactions, Th17 cell differentiation, and lipid and atherosclerosis pathways (Fig. 1T). The DEGs revealed significant changes in genes associated with inflammation, antiviral responses, and lipid metabolism (Fig. 1T). Genes including *ACKR3*, *HSP90AA1*, *ROCK2*, and *SREBF1* showed differential expression, suggesting their involvement in the immune response and lipid metabolism during HEV infection (Fig. 1U).

In conclusion, this study demonstrated efficient suppression of HEV expression using CRISPR-Cas13d. The Cas13d system effectively inhibited HEV infection, reduced liver inflammation and necrosis, and showed potential as a treatment for hepatitis E. The mechanism analysis provided insights into the pathways affected by HEV infection and the impact of Cas13d. It expands the application scope of Cas13d and provides a novel approach to antiviral drug design. The ability to simultaneously target multiple ssRNA viruses using Cas13d and AAV delivery vectors further enhances its potential as an antiviral platform.^5^

## Ethics declaration

Eight-week-old male *Meriones unguiculatus* (∼30 g) gerbils (Laboratory Animal Center, Hangzhou Medical College) were cared for in accordance with the procedures established by the Biomedical Research Ethics Committee of the Chinese Agricultural University (AW01802202-3-1).

## Author contributions

Ran Zhang, Xiaoxiang Hu, and Tianlong Liu conceived, designed, and supervised the study; Chengcheng Zhao, Chong Li, and Pengyao Ren conducted the experiments; Chengcheng Zhao and Sa Li analyzed and summarized the data; and Chengcheng Zhao and Ran Zhang wrote and reviewed the manuscript. All authors reviewed the final version of the manuscript and approved it for publication.

## Conflict of interests

The authors declare that they have no conflict of interests.

## Funding

This work was supported by the National Transgenic Major Program of China (No. 2016ZX08009-003-006) and Plan 111 (No. B12008).

## Data availability

All data and codes in this study are available under proper request via e-mail at zcc987706018@cau.edu.cn.

## Figures and Tables

**figs1:**
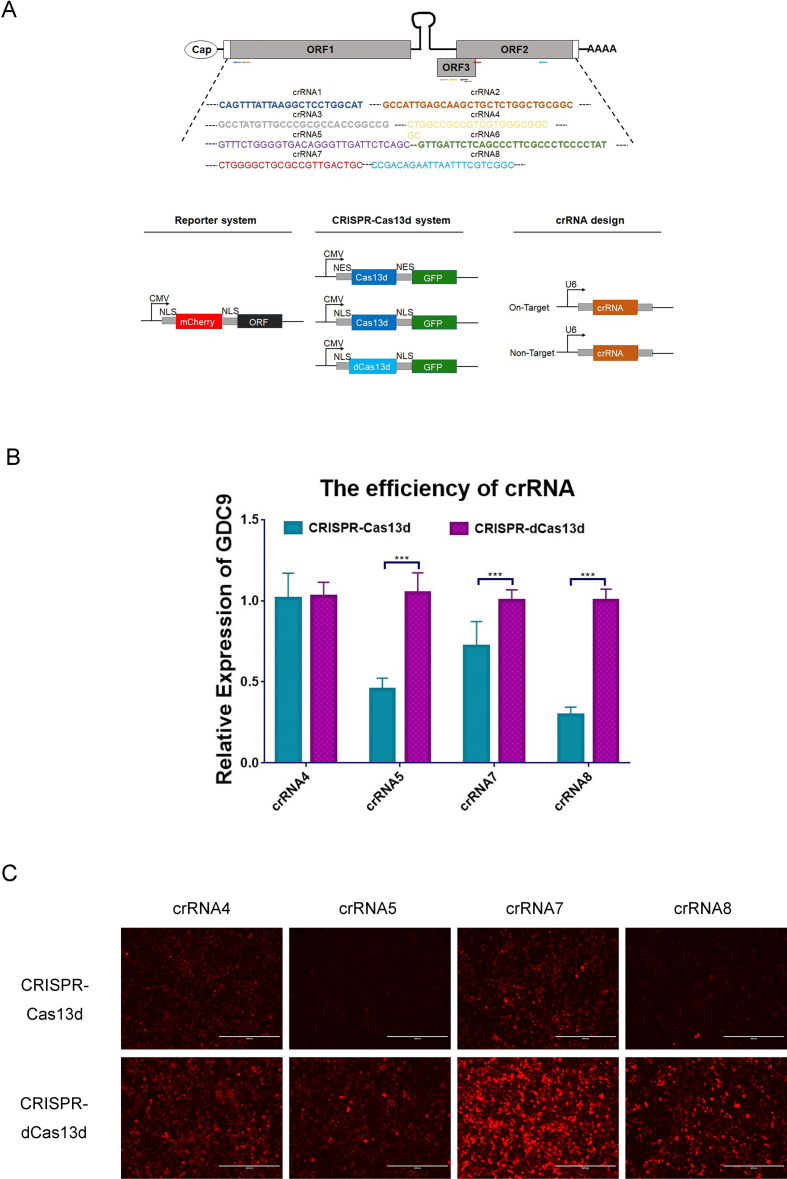


**figs2:**
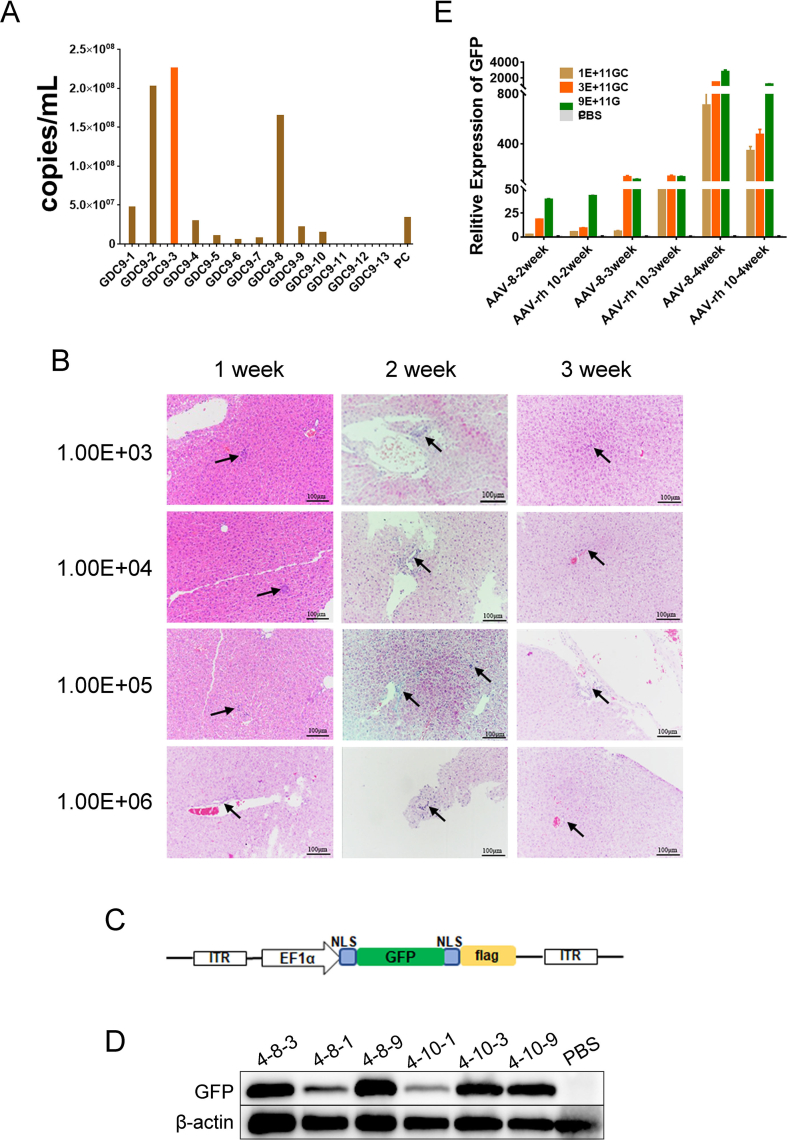


**figs3:**
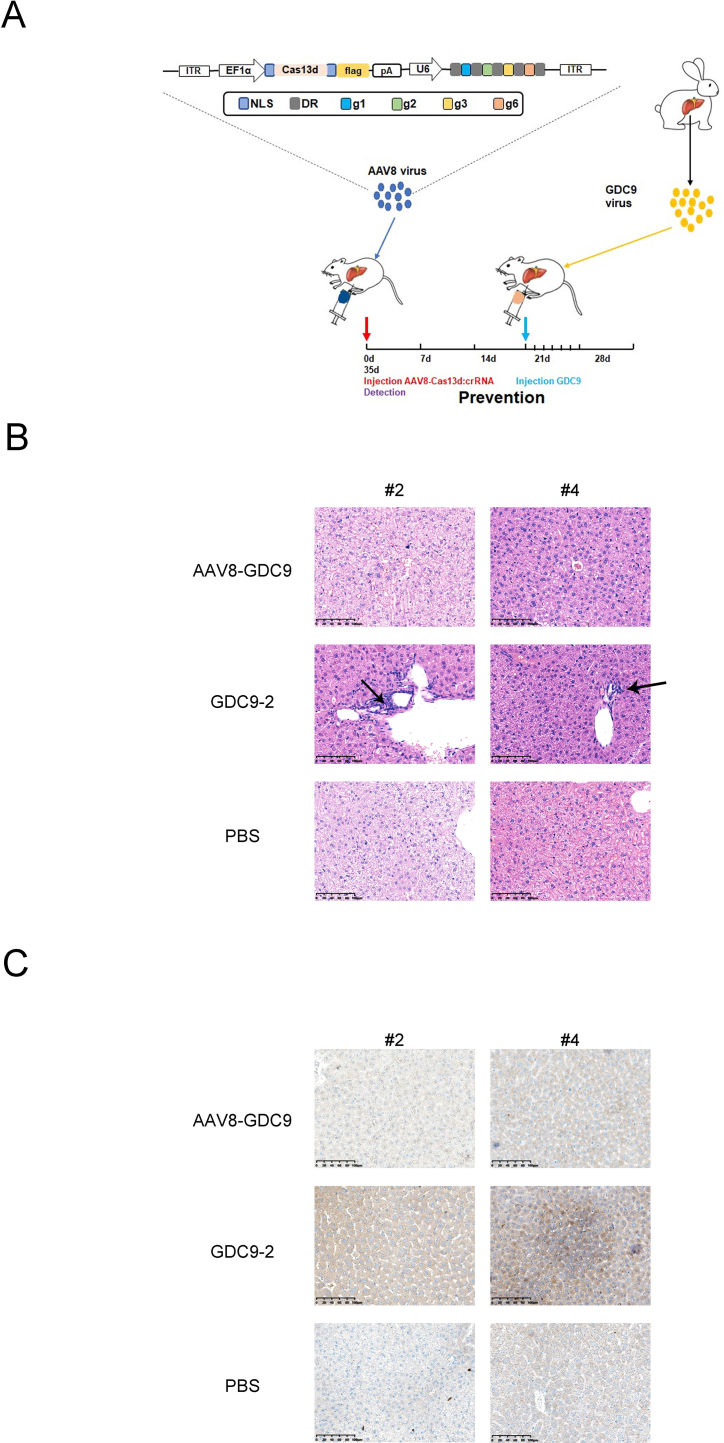


**figs4:**
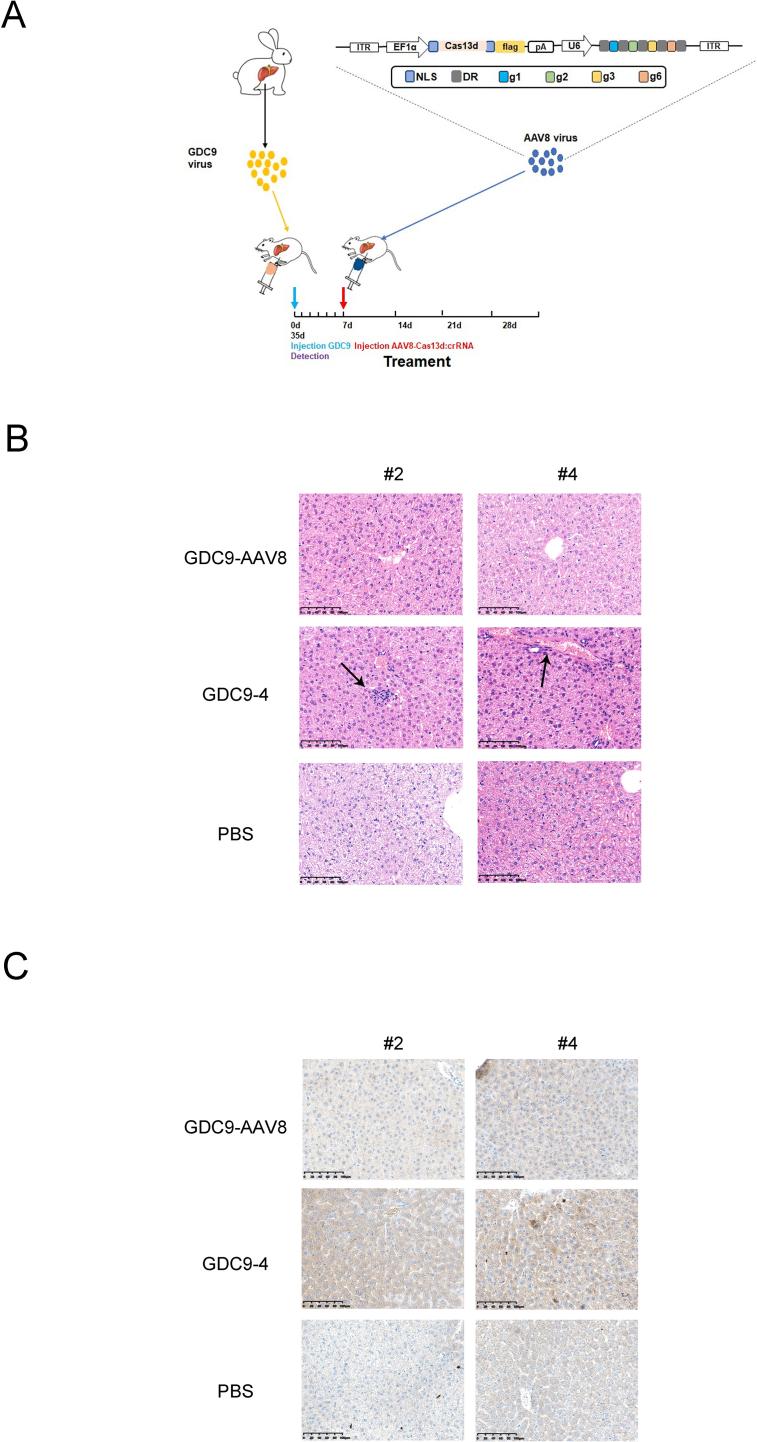


## References

[1] Chaudhry S.A., Verma N., Koren G. (2015). Hepatitis E infection during pregnancy. Can Fam Physician.

[2] Konermann S., Lotfy P., Brideau N.J., Oki J., Shokhirev M.N., Hsu P.D. (2018). Transcriptome engineering with RNA-targeting type VI-D CRISPR effectors. Cell.

[3] Jackson A.L., Bartz S.R., Schelter J. (2003). Expression profiling reveals off-target gene regulation by RNAi. Nat Biotechnol.

[4] Wang Q., Liu Y., Han C. (2021). Efficient RNA virus targeting via CRISPR/CasRx in fish. J Virol.

[5] Naldini L. (2015). Gene therapy returns to centre stage. Nature.

