# Supplementary material for: Prevention and treatment of gerbil hepatitis E using the programmable CRISPR-Cas13d system
Source: Genes Dis. 2023 Jul 24;11(4):101051. doi: 10.1016/j.gendis.2023.06.020 (PMC10950820; doi:10.1016/j.gendis.2023.06.020)
Supplement: Multimedia component 1 [file mmc1.docx]

**Material and methods**

***1 Plasmid construction***

The complete HEV open reading frame (ORF) sequences were synthesized by Sangon. The mCherry expression backbone was digested with BsrGI-HF (NEB, UK) and HindIII-HF (NEB, UK), and then ligated using T4 DNA ligase (NEB, UK).

The chicken β-actin promoter was used to express codon-optimized human Cas13d in a mammalian expression vector with one nuclear localization signal ^15^. The Cas13d/dCas13d-expression plasmids used herein were donated by Prof. Xingxu Huang from the University of Shanghai for Science and Technology.

Cas13d crRNAs (HEV-crRNA1–8, and non-crRNA) were synthesized and ordered as single-stranded DNA oligonucleotides. They were annealed and cloned into an expression vector containing the U6 promoter and BspQI enzyme sites. These ssDNA oligonucleotides are listed in Table 1.

The IP481-ITR-EFS-CasRx-Flag-polyA-U6-DR-sgRNAs-DR-ITR vector was a gift from Prof. Hui Yang, Shanghai Institute for Biological Science, Chinese Academy of Sciences. To insert the four crRNA sequences, tandem crRNAs (HEV-crRNA1, 2, 3, and 6) were synthesized and subcloned into IP481 using SacI and SpeI enzymes (NEB, UK) to generate a single clone.

***2 Cell culture and transfection***

HEK293FT cells were cultured in Dulbecco’s modified Eagle’s medium (DMEM; 4.5 g/L glucose) containing 10% fetal bovine serum (FBS; Gibco, USA) at 37°C with 5% CO_2_. The cells were transfected, and 1 × 10^5^ cells were plated the day after in complete growth medium. To transfect the cells in each well, dilutions of 1.0 μg of DNA and 2 μL of P3000 (Thermo Scientific, USA) in 25 μL of Opti-MEM and 0.75 μL of Lipo 3000 (Thermo Scientific, USA) were used. After mixing, 50 μL of the DNA-Lipo 3000 complex was added to each well, and the plate was gently rocked. Complexes were removed 6 h post-transfection, and complete growth medium with 10% FBS was added. After transfection, the cells were incubated in a 5% CO_2_ incubator at 37°C for 48 h before assessing gene expression.

***3 The cell line***

HEK293FT cells were purchased from the National Biomedical Experimental Cell Resource Bank, Cat. No. 1101HUM-PUMC000091. HEK293FT cells expressed the SV40 T antigen and could be transfected and used as packaging cells. The AP fusion protein was highly expressed when used for transient transfection.

***4 Reverse transcription quantitative*** ***polymerase chain reaction (RT-qPCR)***

After 48 h of transfection, total RNA was extracted according to the Omega RNA purification kit protocol (Omega Bio-tek, USA). Total RNA was reverse-transcribed to synthesize first-strand cDNA using the PrimeScript™ RT reagent kit with a gDNA eraser (Perfect Real-Time; TaKaRa, Japan), as per the manufacturer’s instructions.

Amplification was performed on an ABI 7300 (Applied Biosystems, USA) using TB Green® Premix Ex Taq™ (Tli RNaseH Plus; TaKaRa, Japan). *GAPDH* was used as an internal control. Data are presented as the mean ± SD, and expression levels were normalized to *GAPDH*. The primers used for qPCR analysis are listed in Table 2.

***5 AAV packaging***

AAV packaging was performed by PackGene Biotech. Briefly, AAV plasmids, along with the adenoviral helper as well as AAV rep and AAV8 cap genes, were introduced into HEK293FT cells using Polythylenimine Max (PEI, Polysciences) in DMEM with 10% FBS. The supernatant was harvested seven days later and purified via iodixanol density gradient purification. The viral genomic titer was determined via qPCR (3E+13 GC).

***6 HEV3-GDC9 virus***

The HEV3-GDC9 virus used in this study was a gift from Prof. Tianlong Wang, China Agriculture University ^19^, isolated from Chinese farm rabbits in 2009 ^19^. The complete cDNA of the GDC9 genome was sequenced and confirmed [GenBank: FJ906895.1].

***7 Viral copy detection***

Viral RNA was extracted using the QIAamp Viral RNA Mini Kit according to the manufacturer’s instructions. Complementary DNA was synthesized as described above. Absolute quantification PCR was performed using Premix Ex Taq (Probe qPCR; TaKaRa, Japan), as per the manufacturer’s instructions.

GDC9 copies were determined using cDNA as a template; the primers are listed in Table 3 (ordered from Sangon, China). The primers used to measure GDC9 RNA are listed in Table 4 (ordered from Sangon, China). RT-PCR was performed using RNA as a template and the PrimeScript™ One-Step RT-PCR Kit Version 2 (TaKaRa, Japan), according to the manufacturer’s instructions. The rate of viral release was calculated by dividing the number of virus-excreting gerbils by the total number of gerbils in each group.

Applied Biosystems Analysis^TM^ Software was used for absolute quantification PCR analysis. A standard curve of GDC9 PCR target fragments from 10^11^ to 10 copies per μL was used to quantify the viral RNA copy number in each replicate, with three technical replicates per sample.

***8 Intraperitoneal injection of the gerbils***

Eight-week-old male *Meriones unguiculatus* (~30 g) gerbils (Laboratory Animal Center, Hangzhou Medical College) were cared for in accordance with the procedures established by the Biomedical Research Ethics Committee of the Chinese Agricultural University (AW01802202-3-1). The gerbils received an intraperitoneal GDC9 injection of 200 μL for seven days, and either AAV8 or AAV rh10 was also injected at 200 μL. The gerbils were anesthetized with carbon dioxide before being dissected and sampled.

***9 Western blot analysis***

Total protein was extracted from gerbil liver tissue using RIPA lysis buffer (Cell Signaling Technology, USA) with protease inhibitors. Protein concentration was determined using the bicinchoninic acid method (CWBIO, China). A total of 30 μg of protein was subjected to sodium dodecyl-sulfate polyacrylamide gel electrophoresis (SDS-PAGE) and then transferred to polyvinylidene difluoride membranes. After blocking with 5% non-fat milk, the membranes were washed three times with PBST. Immunoblot analysis was performed with an anti-GFP antibody (1:5000, Abmart, China) and an anti-actin antibody (1:5000, Abmart, China). Chemiluminescence was used to detect the signals (Thermo Scientific, USA).

***10 Histology, immunofluorescence, and immunohistochemistry***

The gerbil livers were dissected, fixed in Bouin’s fluid for 24 h at 25℃, dehydrated, and embedded in paraffin wax. The tissue blocks were sectioned at 5 μm and stained with hematoxylin and eosin. Additionally, the blocks were sectioned at 10 μm and stained using an anti-flag (1:200, Abmart, China) or anti-GDC9 (1:200, Abcam, China) antibody. All antibodies are listed in Table 5.

***11 RNA sequencing (RNA-seq) analysis***

Transcriptome library construction was performed by Gene^+^ and sequencing was carried out on the Illumina Xten platform. After filtering low-quality reads with FastQC (v0.11.8), the reads were aligned to the Mongolian gerbil (v1.0) reference genome using Hisat2 (v2.1.0). All uniquely mapped reads were used to calculate transcripts per million using string tie (v1.3.5). Limma (v3.50.3) was used to screen for differentially expressed genes (DEGs; fold-change > 1 and FDR < 0.05).

***12 Statistical analysis***

We used a Student’s *t*-test with two-tailed unpaired samples and set the threshold at p < 0.05. All statistical analyses were performed using GraphPad Prism 7.

**Figure Legends**

**Figure S1. Rapid screening of virus-targeting efficient gRNAs. (A)** up:Eight crRNAs in HEV3 ORFs. g1 (blue), g2 (orange), g3 (grey), and g6 (green) were selected for subsequent experiments; down: Schematic illustration of mammalian expression constructs encoding for Cas13d and guides. NLS, nuclear localisation signal; crRNA, predicted mature guide RNA with a single 30 nt processed DR and a 21–30 nt spacer sequence. **(B)** mCherry-ORFs mRNA relative expression. Values shown as mean ± SEM with n = 3. p<0.0001. **(C)** mCherry fluorescent expression of HEK293FT cells transfection mCherry-ORFs construct, Cas13d orthologs and crRNAs. Scale bar, 400μm.

**Figure S2. Establishment of HEV infection in gerbils.** (**A**) Absolute quantification of rabbit-derived HEV3-GDC9 copy number in different batches. PC, positive control (virus with known copy number). **(B)** Quantified detoxification after injection of gerbils with different virus doses. **(C)** AAV construct that expresses GFP. AAV was delivered into Mongolian gerbil livers via intraperitoneal injection. **(D)** GFP mRNA relative expression. Data are shown as the mean ± SD (n = 3). **(E)** GFP protein level in Mongolian gerbil livers four weeks after receiving AAV was analyzed via western blotting. 4, four weeks after AAV delivery; 8 and 10, AAV8 and AAV rh10; 1, 3, and 9, the concentration of AAV given to gerbils. AAV8 packing Cas13d:crRNA-GDC9: AAV8 packing Cas13d:crRNA was injected first, then GDC9. GDC9-2: GDC9 injection for only two weeks. GDC9-AAV8 packing Cas13d:crRNA: GDC9 was injected first, then AAV8 packing Cas13d:crRNA. GDC9-4: HEV3 injection for only four weeks. PBS: PBS injection only.

**Figure S3. CRISPR-Cas13d system for the prevention of hepatitis E in gerbils. (A)** Schematic illustration of intraperitoneal injection of AAV8 packing Cas13d: crRNA and GDC9 first, then GDC9. **(B)** The liver histology of Mongolian gerbil injected with or without AAV8 packing Cas 13d: crRNA and GDC9. The black arrowhead indicates vacuoles. Scale bar: 100 μm. **(C)** Representative GDC9 immunohistochemical (IHC) staining of liver sections from Mongolian gerbils injected with or without the AAV8 packing Cas 13d: crRNA and GDC9 (n =4). The darker the attachment color in different group, the higher the virus content. Scale bar: 100 μm. AAV8 packing Cas13d: crRNA -GDC9: AAV8 packing Cas13d: crRNA was first injected, followed by GDC9. GDC9-2: GDC9 injection for only two weeks; AAV8: only AAV8 packing; Cas13d: crRNA; PBS: only PBS injection.

**Figure S4. Pathological assessment of HEV-infected gerbils receiving CRISPR-Cas13d treatment. (A)** Schematic illustration of intraperitoneal injection of the AAV8 packing Cas13d: crRNA and GDC9. Virus was injected first, followed by the AAV8 packing Cas13d: crRNA. **(B)** The liver histology of Mongolian gerbil injected with or without AAV8 packing Cas 13d: crRNA and GDC9. The black arrowhead indicates vacuoles. Scale bar: 100 μm. **(C)** Representative GDC9 immunohistochemical (IHC) staining of liver sections from Mongolian gerbil injected with or without the AAV8 packing Cas 13d: crRNA and GDC9 (n =4). The darker the attachment color in different group, the higher the virus content. Scale bar: 100 μm. Cas13d: crRNA. Injection of GDC9-only did result in such a decrease. GDC9-AAV8 packing Cas13d: crRNA: GDC9 was first injected, followed by AAV8 packing Cas13d: crRNA. GDC9-4: GDC9 injection for only 4 weeks. AAV8: only AAV8 packing; Cas13d: crRNA. PBS: only PBS injection. Note: PBS-2 and PBS-4 in S3B/S4B and S3C/S4C are the same gerbils, so PBS-2 is the same picture and PBS-4 is the same picture.
